# Wild Bitter Melon Exerts Anti-Inflammatory Effects by Upregulating Injury-Attenuated CISD2 Expression following Spinal Cord Injury

**DOI:** 10.1155/2020/1080521

**Published:** 2020-09-30

**Authors:** Woon-Man Kung, Chai-Ching Lin, Chan-Yen Kuo, Yu-Ching Juin, Po-Ching Wu, Muh-Shi Lin

**Affiliations:** ^1^Department of Exercise and Health Promotion, College of Kinesiology and Health, Chinese Culture University, Taipei 11114, Taiwan; ^2^Department of Biotechnology and Animal Science, College of Bioresources, National Ilan University, Yilan 26047, Taiwan; ^3^Graduate Institute of Systems Biology and Bioinformatics, National Central University, Chungli 32001, Taiwan; ^4^Department of Biomechatronic Engineering, College of Bioresources, National Ilan University, Yilan 26047, Taiwan; ^5^Division of Neurosurgery, Department of Surgery, Kuang Tien General Hospital, Taichung 43303, Taiwan; ^6^Department of Biotechnology, College of Medical and Health Care, Hung Kuang University, Taichung 43302, Taiwan; ^7^Department of Health Business Administration, College of Medical and Health Care, Hung Kuang University, Taichung 43302, Taiwan

## Abstract

**Background:**

Spinal cord injuries (SCIs) induce secondary neuroinflammation through astrocyte reactivation, which adversely affects neuronal survival and eventually causes long-term disability. CDGSH iron sulfur domain 2 (CISD2), which has been reported to be involved in mediating the anti-inflammatory responses, can serve as a target in SCI therapy. Wild bitter melon (WBM; *Momordica charantia* Linn. var. abbreviata Ser.) contains an anti-inflammatory agent called alpha-eleostearic acid (*α*-ESA), a peroxisome proliferator-activated receptor-*β* (PPAR-*β*) ligand. Activated PPAR-*β* inhibits the nuclear factor *κ*B (NF-*κ*B) signaling pathway via the inhibition of I*κ*B (inhibitor of NF-*κ*B) degradation. The role of astrocyte deactivation and CISD2 in anti-inflammatory mechanisms of WBM in acute SCIs is unknown.

**Materials and Methods:**

A mouse model of SCI was generated via spinal cord hemisection. The SCI mice were administered WBM intraperitoneally (500 mg/kg bodyweight). Lipopolysaccharide- (LPS-) stimulated ALT cells (astrocytes) were used as an *in vitro* model for studying astrocyte-mediated inflammation post-SCI. The roles of CISD2 and PPAR-*β* in inflammatory signaling were examined using LPS-stimulated SH-SY5Y cells transfected with si-CISD2 or scramble RNA.

**Results:**

WBM mitigated the SCI-induced downregulation of CISD2, PPAR-*β*, and I*κ*B and upregulation of glial fibrillary acidic protein (GFAP; marker of astrocyte reactivation) in the spinal cord of SCI mice. Additionally, WBM (1 *μ*g/mL) mitigated LPS-induced CISD2 downregulation. Furthermore, SH-SY5Y neural cells with CISD2 knockdown exhibited decreased PPAR-*β* expression and augmented NF-*κ*B signaling.

**Conclusion:**

To the best of our knowledge, this is the first study to report that CISD2 is an upstream modulator of the PPAR-*β*/NF-*κ*B proinflammatory signaling pathway in neural cells, and that WBM can mitigate the injury-induced downregulation of CISD2 in SCI mice and LPS-stimulated ALT astrocytes.

## 1. Introduction

Most patients with acute spinal cord injuries (SCIs) exhibit disability. SCIs are associated with expensive and long-term healthcare. The pathophysiology of acute SCIs involves primary and secondary injuries. Primary injury to the spinal cord results in structural damage, disruption of cell membranes and vessels, and degeneration of myelin and axons, which may lead to secondary injury [1]. In SCIs, the pathological mechanisms underlying secondary injuries involve inflammation, free radical production, hyperoxidation, and mitochondrial dysfunction [2, 3]. Extensive injuries to the central nervous system (CNS) may activate the astrocytes, which are the resident immune cells [4, 5]. In response to SCI, astrocytes secrete proinflammatory cytokines and chemokines, thereby inducing a shift in microglial polarization from the beneficial M2 phenotype toward the detrimental M1 phenotype [6–8]. The SCI-induced M1 microglial phenotype is associated with the attenuation of IL-4 expression [9, 10]. The aberrantly activated glial cells (astrocytes and microglia) produce nitric oxide and reactive oxygen species (ROS), which exacerbate the inflammatory response [11]. The activation of inflammatory cascades may be cytotoxic to some neurons and glial cells, which may result in irreversible neurological deficits [12, 13].

Wild bitter melon (WBM) (*Momordica charantia* Linn. var. abbreviata Ser.) belongs to the family Cucurbitaceae. In Asia and Europe, WBM is used as a medicinal herb to treat various pathological conditions, such as inflammation, hyperglycemia, bacterial infection, and oxidative stress [14]. Mice orally administered WBM exhibit upregulated expression of peroxisome proliferator-activated receptor-*α* (PPAR-*α*) and PPAR-*γ* mRNA, which are involved in hypolipidemic and insulin-sensitizing activities [14]. Additionally, WBM inhibits lipopolysaccharide- (LPS-) induced inflammatory responses in macrophages through the regulation of NF-*κ*B activation [15]. Furthermore, WBM extracts have been demonstrated to scavenge free radicals [16], such as 2,2-diphenyl-1-picrylhydrazyl (DPPH) and hydroxyl radicals [17].

The triglycerides in the seed oils of the family Cucurbitaceae comprise conjugated linolenic acid. Alpha-eleostearic acid (*α*-ESA, 18 : 3, *Δ*^9*cis*,11*trans*,13*trans*^, 9*cis*, 11*trans*, 13*trans*-conjugated linolenic acid) is a linolenic acid, which is widely distributed among the members of the family Cucurbitaceae, including WBM and *Momordica charantia* (bitter gourd). *α*-ESA accounts for more than 60% of the total fatty acid composition in bitter gourd seed oil [18]. High-performance liquid chromatography revealed that *α*-ESA accounts for approximately 19% of the total fatty acid composition of the ethyl acetate extracts. The content of *α*-ESA in dried and fresh WBM is 7.1 g/kg and 0.42 g/kg, respectively. In WBM seed oil, *α*-ESA accounts for more than 30% of the total fatty acid content [19].

The ligands of PPAR-*β* (synonyms: PPAR-*δ*) include polyunsaturated fatty acids, such as conjugated linoleic acid [20]. *α*-ESA can be metabolized into conjugated linoleic acid in mice [21] and rats [22]. Therefore, *α*-ESA can be used as a natural ligand for PPAR-*β*. Moreover, PPAR-*β* has been reported to attenuate the production of transforming necrosis factor-*α* (TNF-*α*) in cardiomyocyte culture through the inhibition of the NF-*κ*B (nuclear factor *κ*B) signaling pathway [23]. In a mouse model of bleomycin-induced lung injury, the PPAR-*β* agonist GW0742 inhibited I*κ*B (inhibitor of NF-*κ*B) degradation, thereby consequently deactivating NF-*κ*B [24]. WBM attenuated the generation of inflammatory responses in LPS-stimulated RAW 264.7 macrophages through the inhibition of NF-*κ*B activation [15]. Thus, the interaction between *α*-ESA and PPAR-*β* may inhibit I*κ*B degradation and subsequently attenuate NF-*κ*B activation. The pathological mechanisms underlying traumatic SCIs involve mitochondrial oxidative stress and inflammation. Thus, WBM, which exhibits antioxidant and anti-inflammatory activities, may aid the management of acute SCIs.

CDGSH iron sulfur domain 2 (CISD2), known to be associated with aging, has been reported to be involved in conferring protection against mitochondrial dysfunction-induced inflammatory responses and apoptosis. Previously, we had demonstrated that CISD2 was significantly downregulated under conditions of CNS injury and disease, such as aging mouse brain [25] and hemisection injury to the spinal cord in rats [26]. Injury-induced CISD2 downregulation leads to neuroinflammation and mitochondrial dysfunction. Thus, CISD2 can serve as a potential therapeutic target for SCI. This study is aimed at evaluating the role of CISD2 in mediating the anti-inflammatory effects of WBM and the regulatory effects of CISD2 on the PPAR-*β*/NF-*κ*B signaling pathway using the SCI mouse and *in vitro* cellular injury models.

## 2. Materials and Methods

### 2.1. Extract Preparation and Reagents

WBM dried at low temperature was used to prepare the extract. The WBM samples were ground into a powder and stored at -20°C. WBM powder was incubated with water at room temperature for 24 h with shaking. The suspension was centrifuged at 13,000 g and 4°C for 10 min to remove any residues. The supernatant was freeze-dried, and the lyophilized powder was incubated with ethanol at room temperature for 24 h with shaking. The samples were centrifuged at 13,000 g and 4°C for 10 min, and the supernatant was concentrated under vacuum and stored at -20°C. The concentrated extract was dissolved in absolute alcohol (≧99.8%) before analysis. Absolute alcohol was purchased from Sigma-Aldrich (St. Louis, MO, USA). *α*-ESA purchased from Cayman Chemical (Ann Arbor, MI, USA) was dissolved in absolute alcohol. LPS (obtained from *Escherichia coli* serotype 055:B5) was purchased from Sigma-Aldrich (St. Louis, MO, USA; L-2880).

### 2.2. Animals

Wild-type C57BL/6JNarl mice with an average weight of 22–28 g were obtained from the National Laboratory Animal Center (Taipei, Taiwan). The animals were maintained in a cage for at least 5 days (5 mice per cage) before arrival to our laboratory. The animals had *ad libitum* access to food and water and were maintained under a 12 h dark/light cycle. The experiments were performed according to the guidelines of the Experimental Animal Laboratory. The animal experiments were approved by the Animal Care and Use Committee at National Ilan University, Yilan, Taiwan (ethical approval code: 105-20).

### 2.3. Hemisection SCI in Mice

The animal model of acute SCI was generated as described in our previous study [27]. Briefly, the animals were divided into the following three groups: sham control, SCI, and WBM (*n* = 6/group). The mice were anesthetized using isoflurane and placed in a stereotactic apparatus (David Kopf Instruments, Tujunga, CA) to secure the spinal cord. Posterior spinal decompression was performed under a dissecting microscope by performing laminectomy of the ninth to tenth thoracic vertebra without duraplasty. For hemisection of the spinal cord, the guide of the wire knife was positioned along the vertical plane close to the lateral surface in the lower thoracic vertebra of the spinal cord. The knife was turned medially and then extended 1.5 mm. The guide was lifted 4.0 mm to hemitransect the spinal cord. The sham control group underwent surgery, but hemisection of the spinal cord was not performed. The wound was closed in layers using sutures, and recovery was promoted using a heating pad (36.5°C). The animals were starved for 3 h after surgery. Postoperative care included rehydration using subcutaneous saline injection. The mice were returned to their preoperative housing conditions after surgery. A 5 mm section of the spinal cord with the lesion or a similar area in the sham control group was obtained for mRNA extraction and lysate preparation at 8 or 24 h posthemisection (each time point *n* = 3, total *n* = 6 for each experimental condition).

### 2.4. Treating SCI Mice with WBM

The mice in the WBM group were administered WBM intraperitoneally (500 mg/kg body weight; single dose) immediately after SCI. This dosage has been reported to protect mice against inflammation, oxidative stress [28], and hyperglycemia [29]. The mice in the SCI group were administered normal saline intraperitoneally (500 mg/kg body weight) immediately after SCI. The sham operation group was not administered either saline or WBM.

### 2.5. Neural Cell Lines

The astrocyte cell line (ALT, BCRC 60581) was purchased from the Bioresource Collection and Research Center (BCRC, Hsinchu, Taiwan). ALT cells were cultured in 90% Dulbecco's modified Eagle's medium (DMEM) supplemented with 1.5 g/L sodium bicarbonate and 10% fetal bovine serum (FBS). The SH-SY5Y cell line (ATCC, Manassas, VA, USA), derived from human neuroblastoma cells was cultured in DMEM/F-12 supplemented with 10% FBS in an incubator with an atmosphere of 5% CO_2_ at 37°C.

### 2.6. Treatment of LPS-Stimulated ALT Cells with WBM

ALT cells (1 × 10^6^) cultured in a 35 mm dish were stimulated using 20 *μ*g/mL LPS. Next, the cells were treated with 1 *μ*g/mL WBM or 0.28 *μ*g/mL *α*-ESA for 24 h. Each experiment was performed at least two times with at least three different astrocyte cultures.

### 2.7. Quantitative Real-Time Polymerase Chain Reaction (qRT-PCR)

Total RNA was extracted from the cultured cells using an extraction buffer (TRIzol/phenol/chloroform). The extracted RNA was reverse transcribed into cDNA using oligo-dT and SuperScript II Reverse Transcriptase (Invitrogen, Carlsbad, CA). The cDNAs were subjected to qRT-PCR to quantify the expression of the target genes. The housekeeping gene cyclophilin was used as an internal control. The PCR conditions were as follows: 25 cycles of 94°C for 1 min (denaturation), 55–60°C for 1 min (annealing), and 72°C for 1 min (extension). The primers used for qRT-PCR are shown in Table 1. The qRT-PCR was performed using SYBR Green on an ABI PRISM 7300 HT Real-Time PCR system (Applied Biosystems, Foster City, CA). The minor groove-binding probes and primers for the detection of target genes and cyclophilin were designed by ABI. The threshold cycle (*C*_t_) (the cycle number at which the amount of the amplified target reached a fixed threshold) was determined. The *C*_*t*_ value of the target genes was normalized to that of cyclophilin. Three independent experiments were performed.

### 2.8. RNA Interference

CISD2 was knocked down in SH-SY5Y cells using small interfering RNA (siRNA). The cells were transfected with CISD2-specific siRNA (si-CISD2) or scrambled siRNA (Silencer® Predesigned siRNA; Ambion, Austin, TX) using Lipofectamine™ 2000 reagent (Invitrogen, Carlsbad, CA). The sequences of si-CISD2 were as follows: 5′-GUCCUCUCAUCCUGAAGAATT-3′ and 5′-UUCUUCAGGAUGAGAGGACTT-3′. At 5 h posttransfection, the Lipofectamine 2000-containing medium was replaced with culture medium to allow the cells to recover for 67 h. CISD2 knockdown efficiency was examined using qRT-PCR.

### 2.9. Immunoblotting

Total protein was extracted from the ALT astrocytes and spinal cord tissues using a lysis buffer (20 mM Tris-HCl, 0.1% sodium dodecyl sulfate (SDS), 0.8% NaCl, and 1% Triton X-100). The protein was subjected to gradient electrophoresis on a 12% gel. The resolved proteins were electroblotted onto a nitrocellulose membrane. The membrane was incubated with a blocking reagent. Next, the membrane was incubated with the following primary antibodies at 4°C for 12 h: anti-I*κ*B alpha (E130) (1 : 2000; ab32518; Abcam, Cambridge, MA, USA), anti-PPAR-*β* (1 : 2000; ab23673; Abcam), anti-GFAP (1 : 2000; ab7260; Abcam), anti-*β*-actin (1 : 4000; ab8227; Abcam), and anti-CISD2 (1 : 500; PA5-34545; Thermo Fisher Scientific). The membrane was washed and incubated with goat anti-rabbit IgG (horseradish peroxidase- (HRP-) conjugated secondary antibody) (1 : 5000; 12-348; Merck Millipore) for 1 h. Protein bands were developed using the Immobilon™ Western Chemiluminescent HRP Substrate (WBKLS0500; Merck Millipore). Densitometric analysis of the protein bands was performed using ImageQuant™ LAS 4000 (GE Healthcare Life Sciences).

### 2.10. Cell Viability

The viability of ALT astrocytes was examined using the 3-(4,5-dimethylthiazol-2-yl)-2,5-diphenyltetrazolium bromide (MTT) assay. The cells were seeded in a 96-well microplate for 24 h before use. The WBM- or *α*-ESA-treated ALT cells were incubated with MTT for 4 h. Next, 0.4 N HCl (0.3 mL) in isopropanol was incubated with the mixture overnight to dissolve the formazan crystals. The absorbance of the mixture was measured at 600 nm using an enzyme-linked immunosorbent assay plate reader.

### 2.11. Statistical Analysis

The variables were subjected to the normality test. Variables exhibiting normal distribution were analyzed using the parametric tests, whereas those exhibiting nonnormal distribution were analyzed using the nonparametric tests. The *P* values in the normality test were >0.05. Therefore, parametric tests were used for comparison of means among the experimental groups.

Independent two-sample *t-*tests were used to compare the means of the two groups. One-way analysis of variance was used to analyze the means of more than two groups.

## 3. Results

### 3.1. Effect of WBM and *α*-ESA on the Viability of ALT Cells

**Figure 1:**
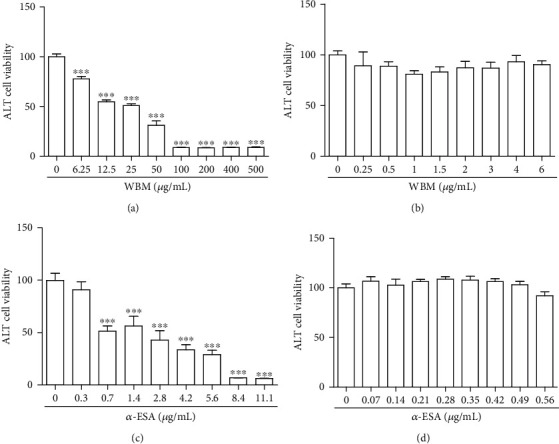
Cell survival, as measured by MTT assay, of ALT astrocytes following treatment with varying doses of WBM (0.25-500 *μ*g/mL) (a) and *α*-ESA (0.07-11.1 *μ*g/mL) (c). Concentrations with less cytotoxic effects were determined (>80% of cell viability) at 0.25-6 *μ*g/mL in WBM (b) and 0.07-0.56 *μ*g/mL in *α*-ESA (d). For *in vitro* experiments, 1 *μ*g/mL WBM and 0.28 *μ*g/mL *α*-ESA were selected. Vertical bars indicate the mean ± standard error of the mean (SEM) (*n* = 3). ^∗∗∗^*P* < 0.001 vs. control. Pair-wise multiple comparisons between groups were determined using the Newman-Keuls method.

The effects of various concentrations of WBM (0.25–500 *μ*g/mL) and *α*-ESA (0.07–11.1 *μ*g/mL) on the viability of ALT cells were analyzed using the MTT assay. Compared with the untreated cells, the WBM- (6.25 *μ*g/mL, Figure 1(a)) or *α*-ESA-treated cells (0.7 *μ*g/mL, Figure 1(c)) exhibited significantly decreased viability. At concentrations of 0.25–6 *μ*g/mL WBM (Figure 1(b)) or 0.07–0.56 *μ*g/mL *α*-ESA (Figure 1(d)), the viability of ALT cells was greater than 80%. Subsequent *in vitro* experiments were performed using 1 *μ*g/mL WBM and 0.28 *μ*g/mL *α*-ESA as at these concentrations, the test agents did not exhibit any cytotoxic effects.

### 3.2. Effect of LPS on the Expression of Proteins Associated with Astrocyte Reactivation and Inflammation

**Figure 2:**
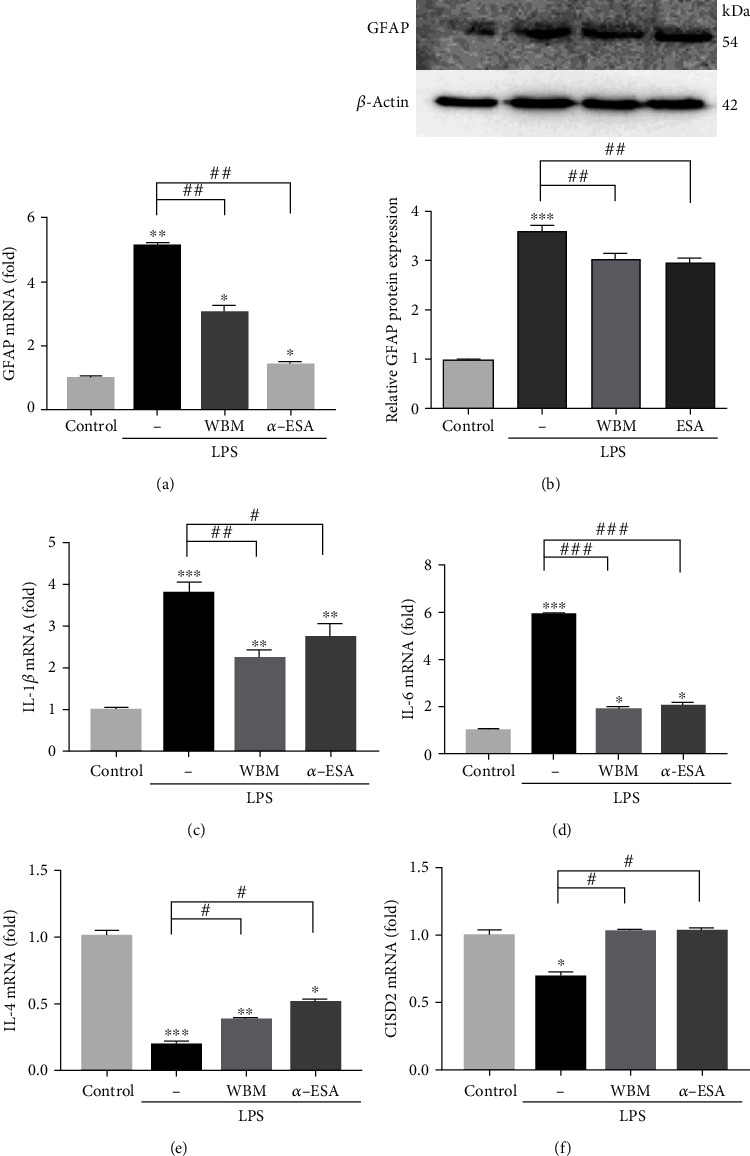
Injury-induced CISD2 downregulation, enhanced GFAP mRNA and protein expression, and proinflammation in LPS-challenged ALT astrocytes. WBM can prevent the abovementioned detrimental effects. Results of mRNA expression of GFAP (a), GFAP protein (b), mRNA expression of IL-1*β* (c), IL-6 (d), IL-4 (e), and CISD2 (f) in ALT cells with or without administration of WBM and *α*-ESA. Vertical bars indicate the mean ± standard error of the mean (SEM) of mRNA expression (*n* = 3). ^∗^*P* < 0.05vs. control,^∗∗^*P* < 0.01vs. control,^∗∗∗^*P* < 0.001vs. control, ^#^*P* < 0.05, ^##^*P* < 0.01, and ^###^*P* < 0.001indicate a significant difference. Pair-wise multiple comparisons between groups were performed using the Newman-Keuls method.

LPS-stimulated ALT cells were used as an *in vitro* model of cellular injuries. This model recapitulates SCI-associated aberrant astrocyte activation and inflammation [25, 26, 30]. The cells were treated with 20 *μ*g/mL LPS for 24 h and subjected to qRT-PCR and western blotting. The expression of GFAP (*P* < 0.01, Figure 2(a)), IL-1*β* (*P* < 0.001, Figure 2(c)), and IL-6 mRNA (*P* < 0.001, Figure 2(d)) was significantly upregulated in LPS-stimulated cells compared with that in the control cells. In contrast, the expression of IL-4 (*P* < 0.001, Figure 2(e)) and CISD2 mRNA (*P* < 0.05, Figure 2(f)) was significantly downregulated in LPS-stimulated cells when compared with that in the control cells. The expression of GFAP in ALT cells was quantified by western blotting. Compared with that in control cells, the expression of GFAP was significantly upregulated in LPS-stimulated ALT cells (*P* < 0.001, Figure 2(b)). These findings indicated that the mechanisms underlying injury-induced secondary damage involve aberrant activation of astrocytes, enhanced inflammatory response, and attenuated expression of IL-4 (potentially resulting in impaired polarization of anti-inflammatory M2 microglia) and CISD2.

### 3.3. WBM and *α*-ESA Attenuated LPS-Induced Changes in ALT Astrocytes

Next, the effects of WBM and *α*-ESA on the expression of IL-4 and CISD2 in LPS-stimulated ALT cells were evaluated. ALT cells were treated with 20 *μ*g/mL LPS, 20 *μ*g/mL LPS, and 1 *μ*g/mL WBM, or 20 *μ*g/mL LPS and 0.28 *μ*g/mL *α*-ESA for 24 h, and subjected to qRT-PCR and western blotting.

Compared with LPS-stimulated ALT cells, ALT cells treated with WBM or *α*-ESA in the background of LPS stimulation exhibited significant downregulation of GFAP mRNA (both *P* < 0.01, Figure 2(a)) as well as protein levels (both *P* < 0.01, Figure 2(b)). Additionally, the expression of IL-1*β* (*P* < 0.01 or *P* < 0.05, respectively, Figure 2(c)) and IL-6 mRNA (*P* < 0.001 or *P* < 0.001, respectively, Figure 2(d)) in ALT cells treated with WBM or *α*-ESA in the background of LPS stimulation was lower than that in LPS-stimulated ALT cells. Moreover, the expression of IL-4 (both *P* < 0.05, Figure 2(e)) and CISD2 mRNA (both *P* < 0.05, Figure 2(f)) in ALT cells treated with WBM or *α*-ESA in the background of LPS stimulation was lower than that in LPS-stimulated ALT cells. These findings indicated that *α*-ESA is the bioactive component, and it is responsible for the anti-inflammatory effect of WBM. We hypothesized that the mechanism underlying the anti-inflammatory activity of WBM in LPS-stimulated ALT cells involves astrocyte deactivation, proinflammatory cytokine attenuation, and enhanced IL-4 and CISD2 expression.

### 3.4. Effect of CISD2 Knockdown on PPAR-*β* Expression and Inflammatory Response in the SH-SY5Y Cells

CISD2 has been reported to inhibit inflammation through the regulation of upstream components of the NF-*κ*B signaling pathway [39]. Attenuation of CISD2 promotes inflammation in various CNS-associated conditions, such as aging [20], injuries, and degeneration [19]. *α*-ESA, a bioactive anti-inflammatory compound in WBM, serves as a PPAR-*β* ligand. Thus, *α*-ESA suppresses NF-*κ*B signaling and downstream cytokine production by inhibiting I*κ*B degradation. Next, the effects of WBM and *α*-ESA on the expression of CISD2 and PPAR-*β*, which are the upstream effectors of the NF-*κ*B signaling pathway, were examined. SH-SY5Y cells are recognized as a well-established *in vitro* model to evaluate neural function. In this study, SH-SY5Y cells were transfected with si-CISD2 to knockdown CISD2. Previous studies have reported that si-CISD2 achieved approximately 60% knockdown efficiency in SH-SY5Y cells [26].

**Figure 3:**
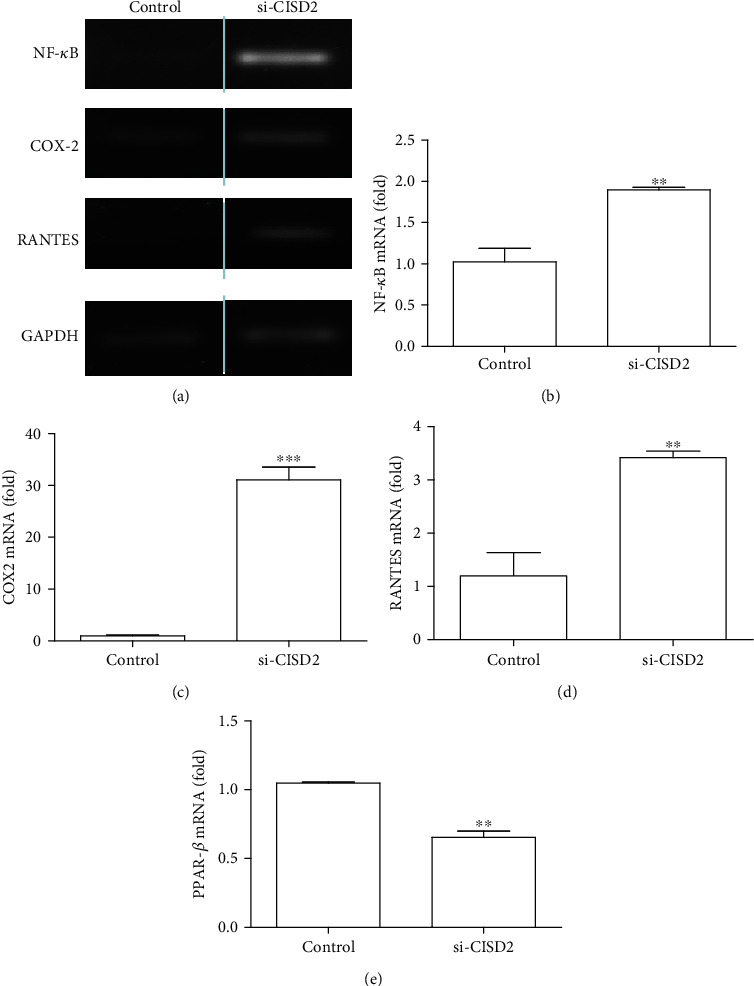
Knockdown of CISD2 expression in neural cells significantly influenced mRNA expression levels in PPAR-*β*, indicating upstream regulation of CISD2 on PPAR-*β* (a). mRNA levels of NF-*κ*B, COX-2, and RANTES were determined by semiquantitative RT-PCR in neural cells with or without si-CISD2 transfection. The results shown are from one of the experiments that were repeated for at least 3 times. Quantification of relative band intensity of semiquantitative RT-PCR showed that CISD2 knockdown in neural cells led to enhanced mRNA expression levels in NF-*κ*B (b) and downstream proinflammatory mediators including COX-2 (c) and RANTES (d). (e) Results of real-time qRT-PCR for mRNA levels of PPAR-*β* in neural cells with or without si-CISD2 transfection. Vertical bars indicate the mean ± standard error of the mean (SEM) of mRNA expression (*n* = 3). ^∗∗^*P* < 0.01 and ^∗∗∗^*P* < 0.001 indicate a statistically significant difference between the control and si-CISD2 groups using independent two-sample *t*-tests.

The results of qRT-PCR are shown in Figure 3(a). The band intensities reproducibly confirmed the anti-inflammatory effect of CISD2. Compared with the scramble siRNA-transfected cells, si-CISD2-transfected cells exhibited significant upregulation of NF-*κ*B p105 (*P* < 0.01, Figure 3(b)), COX-2 (*P* < 0.001, Figure 3(c)), and RANTES mRNA (*P* < 0.01, Figure 3(d)). Furthermore, the expression of PPAR-*β* mRNA in si-CISD2-transfected cells was significantly lower than that in the control cells (untreated with si-CISD2) (*P* < 0.01, Figure 3(e)). These findings indicated that CISD2 acts on the upstream components of the PPAR-*β*/NF-*κ*B signaling pathway, and that it is involved in NF-*κ*B-mediated aberrant activation of astrocytes and proinflammatory cascades.

### 3.5. Effects of WBM on SCI Mice

**Figure 4:**
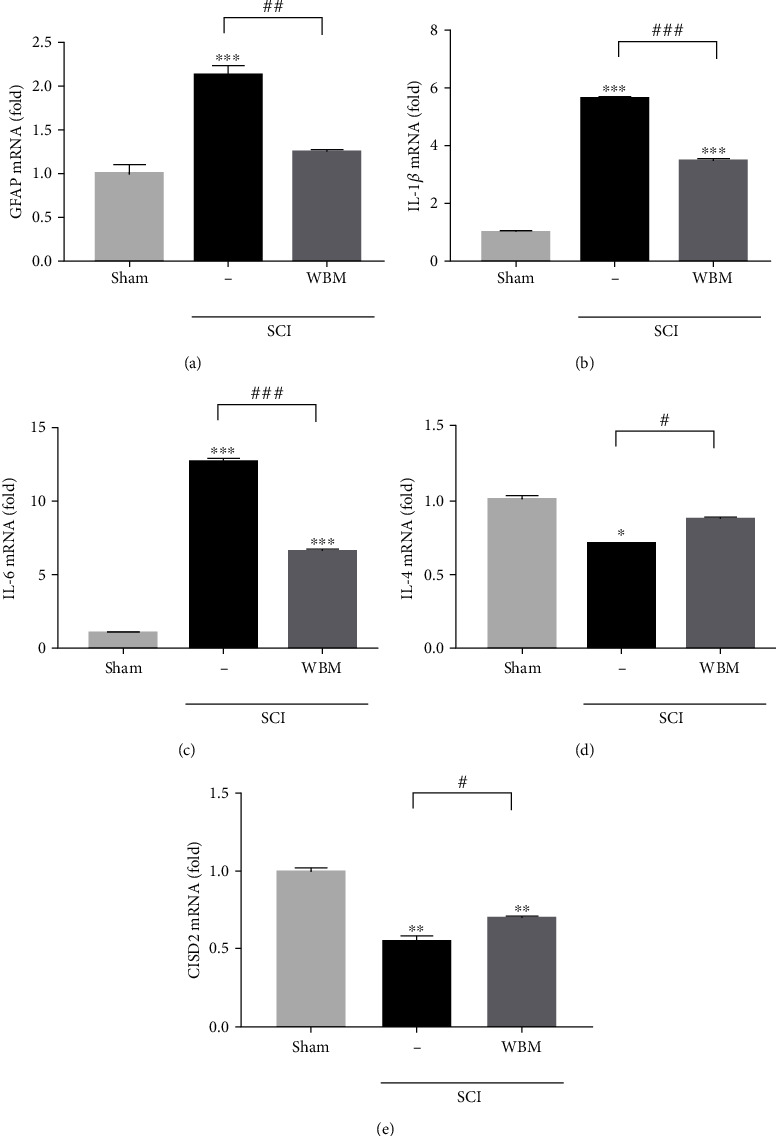
Traumatic insults upregulated GFAP and neuroinflammatory mediators and downregulated CISD2 mRNA expression *in vivo* 24 h after spinal cord hemisection in mice. WBM can *in vivo* rescue the abovementioned detrimental effects. Results of mRNA expression of GFAP (a), IL-1*β* (b), IL-6 (c), IL-4 (d), and CISD2 (e) for 3 conditions. Vertical bars indicate the mean ± standard error of the mean (SEM) of mRNA expression (*n* = 3). ^∗∗^*P* < 0.01 vs. control, ^∗∗∗^*P* < 0.001 vs. control, ^##^*P* < 0.01, and ^###^*P* < 0.001 indicate a significant difference. Pair-wise multiple comparisons between groups were performed using the Newman-Keuls method.

Next, the effect of WBM on the expression of CISD2 in mice with hemisection SCI was evaluated. The SCI mice were intraperitoneally administered WBM (500 mg/kg body weight) or normal saline. At 24 h post-SCI, qRT-PCR was performed to analyze the expression of genes involved in astrocyte-mediated inflammation, such as GFAP, IL-4, and CISD2, in the damaged spinal cord. Compared with the sham control group, the SCI group exhibited upregulation of GFAP (*P* < 0.001, Figure 4(a)), IL-1*β* (*P* < 0.001, Figure 4(b)), and IL-6 mRNA (*P* < 0.001, Figure 4(c)) and downregulation of IL-4 (*P* < 0.05, Figure 4(d)) and CISD2 mRNA (*P* < 0.01, Figure 4(e)). These findings suggested that SCIs result in inhibited IL-4 and CISD2 expression, aberrant astrocyte activation, and enhanced inflammation [26].

Compared with the SCI group, the WBM group exhibited downregulation of GFAP (*P* < 0.01, Figure 4(a)), IL-1*β* (*P* < 0.001, Figure 4(b)), and IL-6 mRNA (*P* < 0.001, Figure 4(c)) and upregulation of IL-4 (*P* < 0.05, Figure 4(d)) and CISD2 mRNA (*P* < 0.05, Figure 4(e)). Thus, the anti-inflammatory effects of WBM in SCI mice may involve inhibition of astroglial activity, attenuation of glia-mediated inflammatory responses, and enhanced production of IL-4 (which potentially enhances the M2 microglial population) and CISD2.

### 3.6. Anti-Inflammatory Mechanisms of WBM in SCI Mice

Finally, we demonstrated that the CISD2/PPAR-*β*/NF-*κ*B signaling pathway is involved in mediating the anti-inflammatory effect of WBM in mice with hemisection SCI. Western blotting was performed to evaluate the therapeutic effects of WBM with respect to the mouse spinal cord with or without SCI.

**Figure 5:**
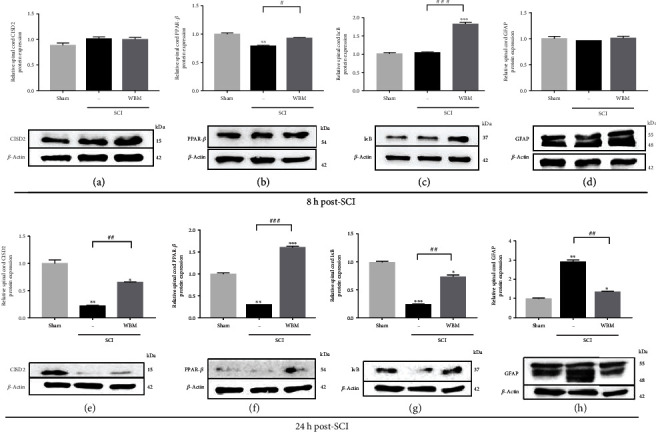
WBM abolished injury-triggered GFAP and attenuated injury-downregulated CISD2, PPAR-*β*, and I*κ*B protein expression in mice 24 h following spinal cord hemisection *in vivo*. (a–d) *In vivo* mouse model of SCI. Results of protein expression of CISD2 (a), PPAR-*β* (b), I*κ*B (c), and GFAP (d) for 3 conditions 8 h after SCI. (e–h) Results of protein expression of CISD2 (e), PPAR-*β* (f), I*κ*B (g), and GFAP (h) for 3 conditions 24 h after SCI. The lower panel indicates representative immunoblot of the proposed protein expression, and *β*-actin (42 kDa) serves as an internal control. The results shown were from one of the experiments that were repeated for at least 3 times. The upper panel indicates the mean ± SEM of the proposed protein/*β*-actin band intensity in ratio to the control group. Anti-inflammatory effects of WBM on SCI can be postulated as CISD2 preservation, and the potential upstream regulation of CISD2 on PPAR-*β*, subsequent I*κ*B stabilization, and therefore, NF-*κ*B inhibition. Vertical bars indicate the mean ± standard error of the mean (SEM) of protein expression (*n* = 3). ^∗^*P* < 0.05 vs. control, ^∗∗^*P* < 0.01 vs. control, ^∗∗∗^*P* < 0.001 vs. control, ^#^*P* < 0.05, ^##^*P* < 0.01, and ^###^*P* < 0.001 indicate a significant difference. Pair-wise multiple comparisons between groups were performed using the Newman-Keuls method.

No detrimental effects were observed at 8 h post-SCI (Figures 5(a), 5(c), and 5(d)). Moreover, the expression of PPAR-*β* (*P* < 0.05, Figure 5(b)) and I*κ*B proteins (*P* < 0.001, Figure 5(c)) in the injured spinal cord of the WBM group was upregulated compared to that in the injured spinal cord of the SCI group. However, WBM-mediated downregulation of GFAP and upregulation of CISD2 in mice with SCI were nonsignificant (Figures 5(a) and 5(d), respectively). The effects of WBM on the expression of GFAP and CISD2 were significant at 24 h post-SCI.

At 24 h post-SCI, compared with the sham group, the SCI group exhibited significant upregulation of GFAP (*P* < 0.01, Figure 5(h)) and significant downregulation of PPAR-*β* (*P* < 0.01, Figure 5(f)), I*κ*B (*P* < 0.001, Figure 5(g)), and CISD2 proteins (*P* < 0.01, Figure 5(e)) in the injured spinal cord. Furthermore, compared with the SCI group, the WBM group exhibited significant downregulation of GFAP (*P* < 0.01, Figure 5(h)) and significant upregulation of PPAR-*β* (*P* < 0.001, Figure 5(f)), I*κ*B (*P* < 0.01, Figure 5(g)), and CISD2 proteins (*P* < 0.01, Figure 5(e)) in the injured spinal cord. These *in vivo* findings suggested that WBM mitigated the SCI-induced downregulation of CISD2 at mRNA and protein levels, and inhibited the PPAR-*β*/I*κ*B/NF-*κ*B signaling pathway, which was in agreement with the *in vitro* results.

## 4. Discussion

Various CNS-associated conditions, such as aging, neurodegeneration, and traumatic brain injury or SCIs are associated with inflammation [31–33] and mitochondrial dysfunction [34, 35]. Activated glial cell-mediated inflammation can contribute to mitochondrial dysfunction, which impairs mitochondrial dynamics and membrane permeabilization and promotes ROS production [36, 37]. Mitochondrial dysfunction can exacerbate the inflammatory response [38]. Prolonged inflammation and mitochondrial dysfunction may lead to irreversible neuronal deficits.

CISD2, an outer mitochondrial membrane protein, is reported to be involved in the maintenance of mitochondrial integrity and calcium metabolism, as well as in the inhibition of apoptosis. It is involved in maintaining the calcium pool in the endoplasmic reticulum (ER), so as to prevent Ca^2+^ surge [39]. Compared with the wild-type mice, the CISD2 knockout mice exhibit increased Ca^2+^ levels in the ER and cytoplasm [40], mitochondrial dysfunction, and enhanced cell death [41]. CISD2 can bind BCL2 and promote the formation of the BCL2-BECN1 complex, which inhibits Beclin 1, a promoter of apoptosis [42].

In addition to mitochondrial dysfunction, CISD2 mitigates CNS injury-induced inflammatory response. Aging, neurodegeneration, and trauma are associated with downregulated CISD2 expression, which enhances the inflammatory response and exacerbates mitochondrial dysfunction [25, 26]. Therefore, therapeutic targeting of CISD2 can aid in preventing the exacerbation of inflammation and mitochondrial dysfunction.

NF-*κ*B signaling is active in the cytoplasm and mitochondrial intermembranous space. The NF-*κ*B signaling pathway is involved in mediating phenomenon, such as mitochondrial dynamics and respiratory electron transport chain. Aberrant activation of NF-*κ*B promotes mitochondrial dysfunction and apoptosis [43]. Additionally, the mechanisms underlying inflammation and mitochondrial dysfunction associated with aging, neurodegenerative disease, and head trauma or acute SCIs may involve aberrant NF-*κ*B activation. Further studies are needed to elucidate the role of CISD2 in regulating the NF-*κ*B signaling pathway.

BCL2 and NF-*κ*B are the upstream targets of CISD2 [44]. In this study, we demonstrated that CISD2 regulates the upstream effectors (PPAR-*β*) of the PPAR-*β*/I*κ*B/NF-*κ*B signaling pathway. Additionally, the findings of this study indicated that the anti-inflammatory mechanism of CISD2 involves inhibition of NF-*κ*B activity. Furthermore, CISD2 is an outer mitochondrial membrane structural protein that may be degraded during the pathological process of primary injury (in acute trauma) or secondary injury (in chronic inflammation) associated with CNS injury or disease. CISD2 is downregulated under nonstressed and injured conditions [25]. Insult-induced CISD2 downregulation results in the inhibition of NF-*κ*B activity in the cytoplasm and mitochondria. This explains the wide range of harmful effects, including enhanced inflammation and mitochondrial dysfunction, associated with injury-induced CISD2 downregulation.

Previously, we had demonstrated that curcumin mitigated injury-induced CISD2 downregulation, suggesting that curcumin exerts a protective effect against inflammation and mitochondrial dysfunction in mice with hemisection SCI [26] and aged mice (104 weeks) [25]. CISD2 deficiency enhances the expression of iNOS and RANTES, which contributes to mitochondrial dysfunctions, such as low DeltaPsi(m) levels, high ROS levels, and augmented apoptosis, which are mitigated upon curcumin treatment [25]. In this study, WBM mitigated the injury-induced downregulation of CISD2, which inhibited the aberrant activation of glia and proinflammatory cascades in SCI-hemisected mice and LPS-stimulated ALT cells. WBM enhanced the expression of CISD2, PPAR-*β*, and I*κ*B proteins in the spinal cord of SCI mice. Hence, we hypothesized that WBM upregulates CISD2, which negatively regulates the PPAR-*β*/I*κ*B/NF-*κ*B signaling pathway and alleviates inflammation and mitochondrial dysfunction through NF-*κ*B. Pharmacological agents that target CISD2 can be considered for treating CNS injury or disease. Further studies are needed to elucidate the role of NF-*κ*B activation in CISD2-mediated alleviation of inflammation and mitochondrial dysfunction.

This study has several limitations. The anti-inflammatory effects of WBM were evaluated based on the expression of CISD2 and attenuation of aberrant glial activation in SCI mice. However, the mechanism underlying injury-induced aberrant glial activation involves astrocyte-microglia interaction. In this study, WBM mitigated the injury-induced upregulation of GFAP in SCI mice and LPS-stimulated ALT cells. This indicated that WBM deactivated the astrocytes. WBM mitigated SCI-induced downregulation of IL-4 in mice with SCI. The loss of IL-4 promotes microglial polarization from M2 to M1 phenotype in IL-4 knockout mice [10]. Thus, WBM can potentially enhance the levels of anti-inflammatory M2 microglia in SCI mice. Further *in vivo* studies are needed to examine the mechanism underlying WBM-mediated microglial polarization in SCIs. Furthermore, si-CISD2 transfection revealed that the anti-inflammatory effects of CISD2 were associated with the regulation of PPAR-*β*, which positively regulates I*κ*B, an inhibitor of NF-*κ*B. Hence, we hypothesized that the injury-induced downregulation of CISD2 inhibited NF-*κ*B activity in the cytoplasm and mitochondria, which resulted in enhanced inflammation and mitochondrial dysfunction. Future studies must elucidate the detailed mechanism underlying CISD2-mediated regulation of NF-*κ*B and glial activation. However, the findings of this study indicated that CISD2 regulates inflammation and mitochondrial dysfunction via the inhibition of NF-*κ*B.

In conclusion, WBM exerted its anti-inflammatory effects through the upregulation of CISD2 in SCI mice and LPS-stimulated ALT cells. CISD2 exhibits protective activity in SCI mice via NF-*κ*B downregulation and astrocyte deactivation.

## Figures and Tables

**Table 1:** Primers.

Gene	Orientation	Sequence
IL-1*β*	Forward	5′-AGGCTCCGAGATGAACAA-3′
	Reverse	5′-AAGGCATTAGAAACAGTCC-3′
IL-4	Forward	5′-TCGGCATTTTGAACGAGGTC-3′
	Reverse	5′-GAAAAGCCCGAAAGAGTCTC-3′
IL-6	Forward	5′-CCACCAAGAACGATAGTCAA-3′
	Reverse	5′-TTTCCACGATTTCCCAGA-3′
GFAP	Forward	5′-CCAACCCGTTCCTCCATA-3′
	Reverse	5′-TCCGCCTGGTAGACATCA-3′
CISD2	Forward	5′-CTTGGAGACTGCTGGGTG-3′
	Reverse	5′-CTTTGCTAAGTCCTCGTC-3′
PPAR-*β*	Forward	5′-GCCGCCCTACAACGAGATCA-3′
	Reverse	5′-CCACCAGCAGTCCGTCTTTGT-3′
NF-*κ*B p105 subunits	Forward	5′-CCAGGGTATGGCTACTCGAACT-3′
	Reverse	5′-GTGACCCCTGCGTTGGATT-3′
COX-2	Forward	5′-ACAAGCACAATAGACGCACAAGA-3′
	Reverse	5′-GGGAGGGCAATTATGATAAGGAT-3′
RANTES	Forward	5′-TGCCCACGTCAAGGAGTATTT-3′
	Reverse	5′-GGCGGTTCCTTCGAGTGA-3′
*β*-Actin	Forward	5′-CTGTCCCTGTATGCCTCTG-3′
	Reverse	5′-ATGTCACGCACGATTTCC-3′
GAPDH	Forward	5′-GGCAAATTCAACGGCACAGT-3′
	Reverse	5′-CGCTCCTGGAAGATGGTGAT-3′

## Data Availability

Answer: yes. Comment: the data used to support the findings of this study are included within the article.
